# Prognostic role of selection criteria for liver transplantation in patients with hepatocellular carcinoma: Review and bibliometric

**DOI:** 10.1016/j.iliver.2024.100077

**Published:** 2024-02-06

**Authors:** Pamela Scarlett Espinoza Loyola, Diana Laura Muratalla Bautista, Karen Adela Hernández Bautista, Elizabeth Gil White, José Antonio González Moreno, Daniel Angel Torres del Real, Víctor Manuel Páez Zayas, Carla Escorza-Molina, Fernando Mondragón Rodríguez, Omar Vásquez Gómez, Luis Jorge Fernández López, Paul Santiago Mogrovejo Vázquez, Isidoro Aczel Sánchez-Cedillo, Víctor Jose Visag Castillo

**Affiliations:** a Social Service Internship, Universidad Nacional Autónoma de México, Mexico City 04510, Mexico; b Social Service Internship, Universidad Anahuac, Mexico City 04510, Mexico; c Department of Surgery Division of Transplantation Surgery, Hospital General de México, Mexico City 06720, Mexico; d Departament of Gastroenterology, Division of Hepatology, Hospital General de México, Mexico City 06720, Mexico; e Departmanet of Anesthesiology, Hospital General de México, Mexico City 06720, Mexico

**Keywords:** Hepatocellular carcinoma, Liver transplantation, Survival, Criteria, Recurrence, Bibliometrics

## Abstract

Hepatocellular carcinoma (HCC) is one of the most common cancers in the world and is one of the leading indications for liver transplantation, liver transplantation is the gold standard treatment for end stage liver disease. Diagnosis is based up on radiological characteristics and rarely biopsy results. However treatment must be individualized to each patient to improve recurrences and outcomes. In this article, we focus on the different selection criteria for liver transplantation. This study aimed to investigate the distribution laws and research frontiers of international literature, so as to present holistic bibliometric evaluation of the studies on 5-year survival and disease-free recurrence in 5 years, according to hepatocarcinoma criteria for liver transplantation. The paper aims to review and analyze 5-year survival and disease-free recurrence based on hepatocarcinoma criteria for liver transplantation. It systematically examines and summarizes distribution characteristics and research frontiers through bibliometric analysis. A bibliographic search was implemented in PubMed/Medline, Clinical Key, Science Direct and Index Medicus with MESH terms, from the year 1996–2022. Patients selected for transplantation using the Metroticket 2.0 (MT2) criteria had the highest overall survival along with patients selected for transplantation using the Milan Criteria had the best 5-year disease-free recurrence. The Metroticket 2.0 criteria (MT2) and Milan Criteria (MC) have shown the most favorable post-transplant outcomes for patients with hepatocellular carcinoma (HCC). However, MC demonstrated the best 5-year disease-free recurrence rate, underscoring the significance of taking into account tumor morphology and biology when determining the eligibility of HCC patients for liver transplantation. The distribution characteristics and research frontiers by bibliometrics concerning prognostic role of selection criteria for liver transplantation in patients with hepatocellular carcinoma the collaborations are sufficient to reach a consensus that the Milan criteria are the best criteria.

## 1 Introduction

Hepatocellular carcinoma (HCC) stands as the fifth most prevalent cancer globally and the second leading cause of cancer-related deaths [[1], [2], [3], [4]]. Its incidence is on the rise in western countries, commonly manifesting in the context of chronic liver disease and cirrhosis. Due to more stringent surveillance programs, early diagnosis has become feasible, leading to improved curative treatments and enhanced survival rates. While various staging and treatment modalities exist for HCC, surgical resection remains the primary treatment. However, a significant proportion of patients are ineligible due to tumor extent or compromised liver function. The Barcelona Clinic Liver Cancer (BCLC) staging system is widely adopted by hepato-pancreato-biliary surgery and liver transplantation programs to guide treatment decisions, considering the limitations imposed by underlying liver disease. Liver transplantation (LT) may be a viable option for a selected group of patients. This paper aims to analyze the 5-year survival and disease-free recurrence within a 5-year timeframe, as reported in the literature, based on hepatocarcinoma selection criteria for liver transplantation.

## 2 Method

### 2.1 Data source and retrieval strategies

**Fig. 1:**
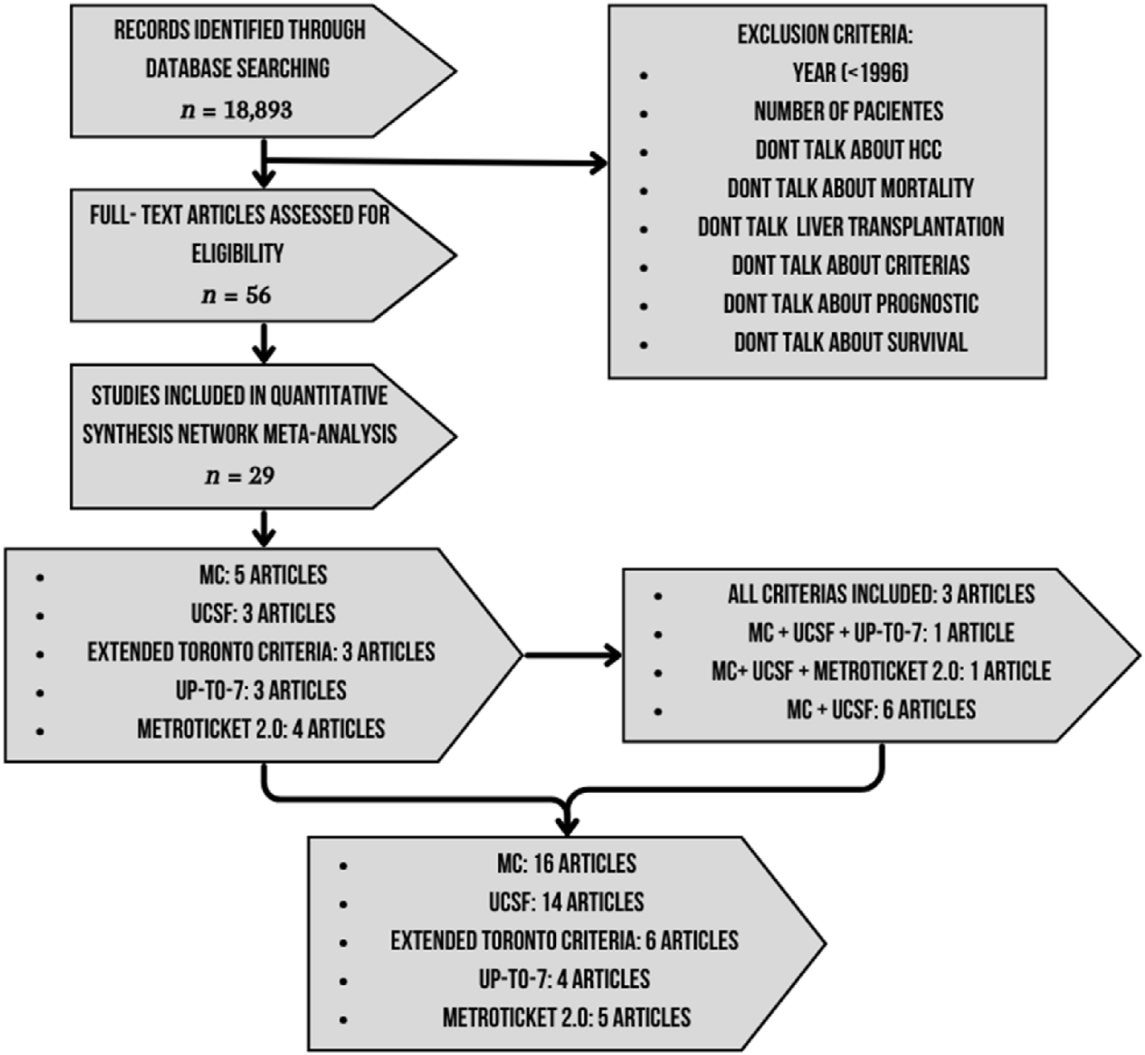
Data retrieval strategies and inclusion procedure of this study.

A bibliographic search was implemented in PubMed/Medline, Clinical Key, Science Direct and Index Medicus with MESH terms, from the year 1996–2022. We included articles evaluating 5-year survival as well as 5-year disease relapse for the different criteria for liver transplantation secondary to hepatocarcinoma; these include the Milan Criteria (MC), University of California, San Francisco (UCSF), Toronto Criteria (ETC), Up-to-7 and Metroticket 2.0 calculator (MT2), The detailded data retrieval strategies and inclusion procedure of this study are shown in Fig. 1.

### 2.2 Bibliometric indicators and visualization

Microsoft Excel (version.2019); was conducted to analyze and draw the leading journal messages, such as annual publication, total number of citations and impact factor et al. In addition, we used Excel to make the world distribution map of the number of articles issued by countries.

### 2.3 Epidemiology

HCC is the most frequent malignant liver tumor, being the 5th most frequent cancer in men and the 7th in women worldwide [5]. In most of cases it occurs as a complication of chronic liver disease (70%–90%) [6]. Cirrhosis is an important risk of factor for HCC; among the different causes are chronic viral hepatitis, alcohol, non-alcoholic fatty liver disease [7,8].

In Mexico, being the 9th cancer, with a similar distribution in both sexes; mortality ranks 3rd, being 3rd in female and 4th in male. The main causes of cirrhosis are alcohol and HVC [9].

### 2.4 Etiology

Approximately 80% of patients diagnosed with HCC have underlying liver cirrhosis of viral etiology (HBV 3%–8%/year and HCV 3%–5%/year) [9].

Chronic infections of HBV causing approximately 55% of all HCCs worldwide, chronic infections with the virus is implicated in the genesis of as many as 85% of the HCCs that occur with such high frequency in Asia and Black African population, in America is <25%; the odds ratio of HCC developing in an individual infected with HBV having been calculated to be 20.4 (males) and 15.6 (females).

HCV is the most common underlying liver disease in North America, Europe and Japan 83%, in Asia <30%. The odds ratio of HCC developing in an individual infected with HCV has been calculated to be 11.5 [10].

However, there are other potential risk factors for HCC such as alcoholic cirrhosis with an annual risk of 1% [11]. The metabolic syndrome the rates are 41%–54% in Mexico [12]; relative risk for HCC to be 1.17 (95% CI: 1.02–1.34) in those were overweight (body mass index [BMI] 25–30 kg/m^2^) and 1.89 (95% CI: 1.51–2.36) in those who were obese (BMI 30 kg/m^2^); obesity and diabetes are also risk factor for HCC in patients with non-alcoholic steatohepatitis (NASH) and fatty liver disease (FLD); 20% of patients with NASH progress to liver fibrosis and ultimately in 3% cirrhosis, NASH and cirrhosis have a risk of developing HCC, which is as high as 12.8% over a 3.2 year median follow-up period [[10], [11], [12], [13]].

Abuse of alcohol, a prevalence five times higher than that of chronic HCV infection. Chronic alcohol abuse (consumption in excess of 80 g/day) such abuse for more than 10 years increases the chance of HCC development approximately fivefold. Meta-analysis has shown a dose–response relationship between alcohol intake and HCC with relative risks of 1.19 (95% CI: 1.12–1.27), 1.40 (95% CI: 1.25–1.56), and 1.81 (95% CI: 1.50–2.19) with intakes of alcohol of 25 g, 50 g, and 100 g per day, respectively [10].

Other risks factors for HCC include: hemochromatosis, biliary disease such as primary cholangitis and primary biliary cholangitis, hepatocellular adenomas (mutation in β-catenin [exon 3] has the risk for malignant transformation 7%) [[10], [11], [12], [13], [14]]. Some toxins such as aflatoxin (AFB1) produced by Aspergilus flavus can be associated to HCC, the correlation between the degree of exposure to AFB1 and the incidence in humas is direct, with odds ratio for developing the tumor of 6.37:1.0 (range 3.74:1.0 to 10.86:10); finally tobacco use is an independent risk factor; has an odds ratios of 1.7 (95% CI: 1.0–2.9) in current smokers compared with nonsmokers and 1.56 (95% CI: 1.29–1.87) in current smokers compared with never-smokers98 have been documented, as has a ratio of 1.49 (95% CI: 1.06–2.10) in former smokers compared to never-smokers [[10], [11], [12], [13], [14], [15]].

### 2.5 Biology

It is highly refractory to most systemic therapies. Recently, significant progress has been made in uncovering genomic alterations in HCC, including potentially targetable aberrations. The most common molecular anomalies in this malignancy are mutations in the TERT promoter, TP53, CTNNB1, AXIN1, ARID1A, CDKN2A and CCND1 genes. PTEN loss at the protein level is also frequent. Genomic portfolios stratify by risk factors as follows (I) CTNNB1with alcoholic cirrhosis; and (II) TP53 with hepatitis B virus-induced cirrhosis. Activating mutations in CTNNB1 and inactivating mutations in AXIN1 both activate WNT signaling. Alterations in this pathway, as well as in TP53 and the cell cycle machinery, and in the PI3K/Akt/mTor axis (the latter activated in the presence of PTEN loss), as well as aberrant angiogenesis and epigenetic anomalies, appear to be major events in HCC. Many of these abnormalities may be pharmacologically tractable. Immunotherapy with checkpoint inhibitors is also emerging as an important treatment option [15].

### 2.6 Morphology

Historically, hepatocellular carcinomas were largely thought of as a single homogenous tumor, but over time many different layers of heterogeneity were recognized at the levels of etiology, clinical presentation, the degree of fibrosis in the background liver, radiology findings, H&E morphology, molecular studies, and outcomes [16].

#### 2.6.1 Macroscopic heterogeneity

Small hepatocellular cellular carcinomas are defined by a diameter less than 2 cm. They have been classified into two major subtypes based on whether the hepatocellular carcinoma is well circumscribed and stands out from the background liver (distinctly nodular) or does not (vaguely nodular). The vaguely nodular pattern is thought to develop from dysplastic nodules and frequently has a few residual portal tracts. Vaguely nodular hepatocellular carcinomas are almost always seen in livers with advanced fibrosis/cirrhosis (>90%) and they tend to be well differentiated (>90%). They typically do not have a well-developed pseudocapsule, are hypovascular on imaging, and only rarely have vascular invasion (5%). In contrast, the distinctly nodular form of small hepatocellular carcinoma is more likely to have a pseudocapsule (50%), is more often moderately differentiated (60%), is hypervascular on imaging, and is more likely to have vascular invasion (40%). Additional macroscopic growth patterns have been described for hepatocellular carcinomas. The nodular type and the massive type grow as distinct nodules, with or without smaller satellite nodules. The nodular type can be of any size, while the massive type replaces almost an entire lobe of the liver. Pedunculated hepatocellular carcinomas grow as mass lesions protruding from the capsule surface. Diffuse hepatocellular carcinoma grows as a proliferation of small tumor nodules that have a similar size and shape to cirrhotic nodules (synonym is cirrhotomimetic). With diffuse hepatocellular carcinoma, imaging and gross examination frequently underestimate the extent of the tumor, as the tumor nodules closely mimic ordinary cirrhotic nodules. Of the different macroscopic growth patterns, only the diffuse pattern is routinely classified as a distinct hepatocellular carcinoma subtype [16].

#### 2.6.2 Microscopic growth patterns

There are four principal microscopic growth patterns (Table 1): trabecular (approximately 70% of tumors have this as the predominant growth pattern), solid (20%; synonym is compact), pseudoglandular (10%; synonym is pseudoacinar), and macrotrabecular (1%).

**Table 1:** Adapted from Torbenson MS [16]. Hepatocellular carcinoma subtypes by order of frequency.

Subtype	Frequency (%)	Prognosis*	Unique histology	Unique clinical findings**	Unique molecular findings***
Steatohepatitic	20	Similar	Yes	Risk factor of fatty liver disease	No
Macrotrabecular massive	10	Worse	Partial	Elevated serum AFP	No
Clear cell	7	Better	Yes	No	No
Scirrhous	5	Similar to better	Yes	No	No
Chromophobe	5	Similar	Yes	No	Yes
Cirrhotomimetic	1	Worse	Partial	No	No
Fibrolamellar carcinoma	1	Similar to better	Yes	Young age, no underlying liver disease	Yes
Granulocyte-colonystimulating-factor producing	<1	Worse	Yes	Elevated serum white blood cell counts, IL6, CRP	No
Lymphocyte rich	<1	Better	Yes	No	No
Lymphoepithelioma hepatocellular carcinoma	<1	Insufficient data	Yes	No	No
Combined tumors					
Hepatocellular-cholangiocarcinoma	1	Worse	Yes	Both AFP and Ca19-9 can be elevated; Higher risk for LN metastases	No
Hepatocellular and neuroendocrine	<1	Worse	Yes	No	No
Carcinosarcoma	<1	Worse	Yes	No	No

*Note*: * Compared to conventional hepatocellular carcinoma; ** Other than prognosis; *** Sufficiently unique to help define the subtype.

#### 2.6.3 Hepatocellular carcinoma subtypes

Overall, about 70% of all hepatocellular carcinomas can be subclassified into morphological subtypes (Table 1). At this time, there are 13 fairly well-established hepatocellular carcinoma subtypes [16].

### 2.7 Pathogenesis

Development of hepatocellular carcinoma is a complex multistep process that involves sustained inflammatory damage, including hepatocyte necrosis and regeneration, associated with fibrotic deposition. Risk of hepatocellular carcinoma emerges when cirrhosis is established, and it increases in parallel to progressive liver function impairment. Hepatocellular carcinoma is the result of the accumulation of somatic genomic alterations in passenger and driver genes in addition to epigenetic modifications, which explains its huge molecular heterogeneity [6].

### 2.8 Diagnosis

Since the diagnosis is made on the basis of cirrhotic patients the aim of surveillance is to improve outcomes and decrease mortality rates, the most used tests for screening are alfafetoprotein (AFP) over 500 ng/mL or an increase of 15 ng/(mL·month)^−1^ and liver ultrasound nodule (s) ≥ 10 mm lesion (US) the sensitivity is 87% and 75% respectively when used alone. Both are screening tests [17].

The diagnosis of HCC is based on imaging techniques/or biopsy, in 2011, the American College of Radiology (ACR) published a system for reporting liver CT and MRI in patients with cirrhosis or other risk factors for HCC, the LIRADS (Liver Imaging Reporting and Data System). Imaging findings are assigned to one of five categories ranging from LR1 (definitely benign) to LR5 (definitely HCC the characteristic image on a single dynamic contrast-enhanced technique (CT) shows intense arterial uptake followed by ‘‘washout’’ of contrast in the venous delayed phases makes the diagnosis) [18].

As the patient undergo for initial evaluation with contrast-enhanced (CT), this also serves to rule out extrahepatic spread or macrovascular involvement, which could contraindicate transplantation [19].

### 2.9 Staging systems

Once HCC is diagnosed, the next step is staging. Among many staging systems developed worldwide the BCLC staging system is the most useful to guide treatment decision [20].

**Fig. 2:**
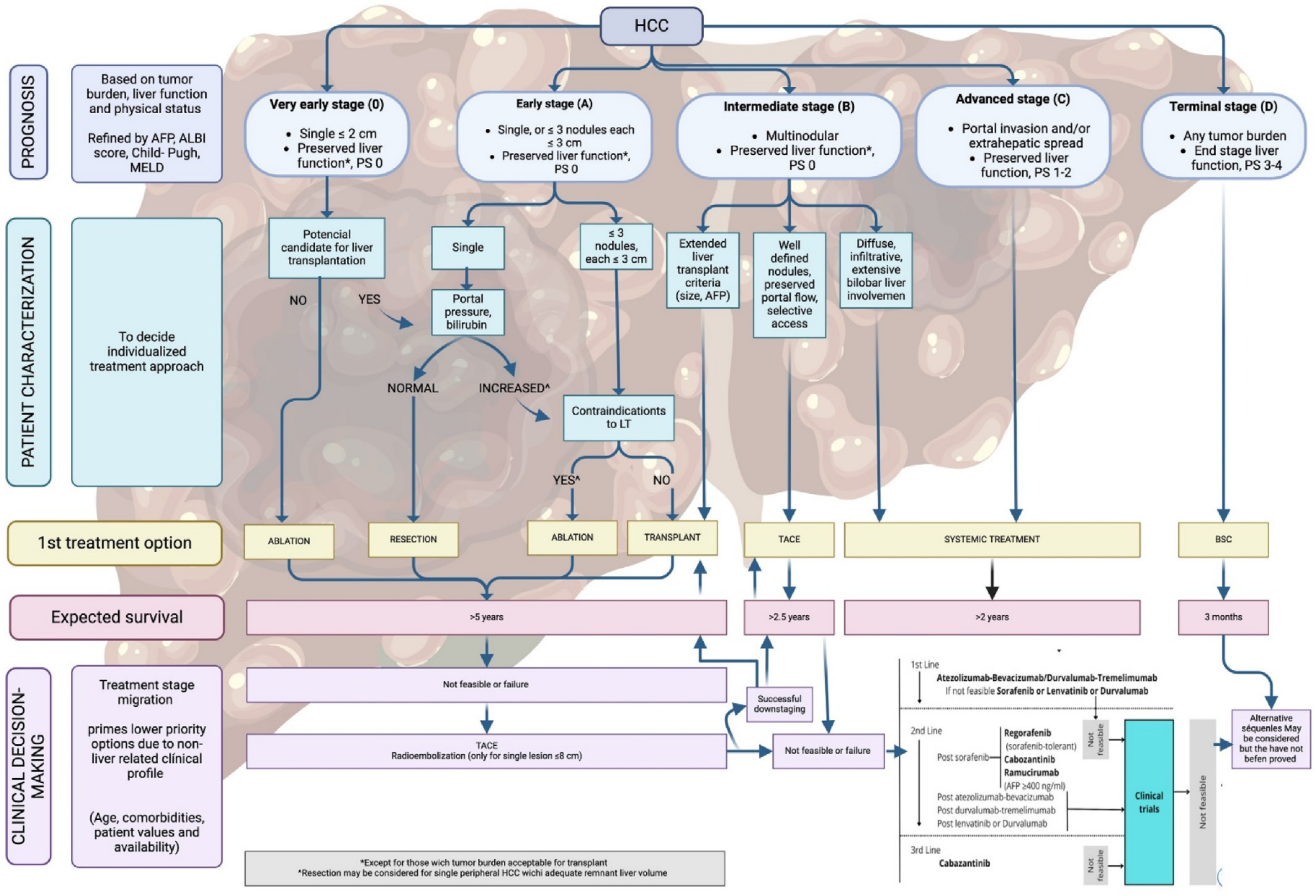
Adapted to Barcelona Clinic Liver Cancer staging and treatment strategy in 2022 [20]. AFP, alpha-fetoprotein; ALBI, albumin-bilirubin; BCLC, Barcelona Clinic Liver Cancer; BSC, best supportive care; ECOG-PS, Eastern Cooperative Oncology Group-performance status; LT, liver transplantation; MELD, model of end-stage liver disease; TACE, transarterial chemoembolisation.

According with published results, this staging system allows an estimation of life expectancy. One of the most useful characteristics of this system is to diagnose of patients with early HCC potentially curable, identify those that may benefit from LT (tumor that is not suitable for surgical resection: inadequate hepatic reserve, tumor location and extent of disease) [21] (Fig. 2).

### 2.10 Therapeutic options

Therapeutic options for HCC includes (Table 2): Down-staging: Local regional therapy (LRT) is typically used as a bridge to control tumor growth and reduce the risk for wait-list dropout, with tumor progression despite LRT associated with worse post-LT outcome [22].

**Table 2:** Adapted from Reig et al. [22]. Therapeutic options for HCC.

THERAPEUTIC OPTIONS FOR HCC			
TYPE	INDICATION	ADVANTAGES	DISADVANTAGES
RADICAL TREATMENT			
Resection	• BCLC A • No portal hypertension • Suitable tumor location • Adequate liver reserve • Suitable liver remnant [19]	• Can use in healthy liver, liver disease and cirrhotic liver (depending on remaining liver volume) • Survival in 5- year >70% • Disease-free recurrence in 5 years > 50% [19,20]	• Operative mortality • Inadequate liver reserve • Acute liver failure • Postoperative decompensation [20]
Liver transplantation	• BCLC A • Treatment for both HCC and cirrhosis • No portal hypertension • Suitable tumor location • Adequate liver reserve • Suitable liver remnant [19]	• Survival in 5- year >75% [20]	• Limited availability of organs • Long wait time [20]
LOCOREGIONAL THERAPY			
Radiofrequency ablation (RFA)	• BCLC 0-A • Tumor <2 cm (No candidates for resection) [19]	• Higher ablative capacity • Fewer sessions than the PEI • Availability • Similar efficacy to surgical resection • Survival in 5-year 76% [20]	• Higher cost than PEI • Higher adverse events than PEI • Less applicability (localization of tumour) • Limited efficacy in tumors in contact with blood vessels [20]
Trans-arterial chemoembolization (TACE)	• BCLC B • Single nodule <5 cm or multifocal (except in multicentric involvement of both liver lobes) HCC [19]	• Compensated liver disease • Partial response 15%–62% • Survival in 1-year 70% [20]	• Post embolization syndrome • Down-staging [20]
SYSTEMIC TREATMENT			
Sorafenib	• BCLC C • Portal invasion/or extrahepatic spread [19]	• Improve survival • Survival: 10.7 months/13.6 months [20]	• Adverse effects • Higher risk of developing adverse events [20]
Lenvatinib	• BCLC C • Portal invasion/or extrahepatic spread [19]	• Improve survival • Survival: 13.6 months [20]	• Adverse effects • Higher risk of developing adverse events [20]

Surgical resection is the best curative treatment of HCC without underlying liver disease (depends on remnant liver volume) offering a 5-year survival rate of 60%–70%. However in this review we will focus on LT [23].

### 2.11 Liver transplantation

Liver transplantation is an option in patients with cirrhosis, end stage liver disease and HCC, because in the same procedure the tumor can removed meanwhile replacing the cirrhotic liver without the risk of poor remnant liver volume as in a surgical resection. Over the years, different criteria have been developed to establish, depending on the characteristics of the tumor, who benefits from a liver transplant, also, LT provides significantly better survival rates than palliation for patients with selected advanced HCC [24].

#### 2.11.1 Milan criteria (MC)

In 1996, Mazaferro et al. assessed 48 patients with HCC who presented for liver transplantation at a single center (Milan, Italy) over a 3-year span. Among their cohort, 60 (20.3%) had histologically proven cirrhosis [25]. The Milan criteria state that transplantation should be performed in those with a single tumor of 5 cm or less or three tumors that are each 3 cm or less, no macrovascular invasion, and no metastasis (Table 3). LT offers in these cases the possibility of cure the tumors should meet the Milan Criteria.

**Table 3:** Adapted from  Ruch et al. [26]. Model based on level of AFP, tumour size and tumour number, to determine risk of death from HCC-related factors after liver transplantation.

STUDY	BASIS	No OF PATIENTS	TYPE	TISSUE/BIOPSY NEEDED?	FINDINGS
Milan Criteria (Mazaferro 1996)	Imaging	48	Largest tumor <5 cm, or • No more than 3 tumor nodules, each <3 cm, and • No obvious vessel or nodal involvement.	No	4- year survival: 75% (35 patients) [27,28,29] 4-year recurrence-free survival: 83% (39 patients) [27,28] and 92% (44 patients) [29]
UCSF (Yao 2001)	Imaging	70	Single tumor ≤6.5 cm, or • ≤ 3 tumors • Largest ≤4.5 cm diameter and • Total tumor diameter ≤8 cm	No	5-year survival: 75.2% (52.6 patients) [28] and 87% (60.9 patients) [26], 72% [30] 5- year recurrence- free survival: 88.6% (62 patients) [26], 75% (50.4 patients) [30]
Up-To-7 (Mazaferro 2009)	Imaging	1556	Sum of the diameter (cm) of largest tumor and the number of tumors ≤7 • Excluded microvascular invasion	Yes	5-year survival: 71.2% (283 patients) [27,28,29] 5- year recurrence- free survival: 64.1% (997.3 patients) [31]
Extended Toronto (DuBay 2011)	Imaging	105	• No tumor size or nomber limit • No systemic spread or vascular invvolvement • Not poorly differentiated on biopsy (if exceeds Milan)	Yes	5-year survival: 68% (71.4 patients) [28,32] 5-year recurrence-free survival: 66% (69.3 patients) [33,28,29]
Metroticket 2.0 (Mazaferro 2018)	Imaging and static AFP	1359	• Up-to-7 criteria • Last pre-op AFP (<200; 200–400; 401–1000; >1000 ng/mL)	No	5-year overall survival: 79.7% (1240 patients) [28], 78% (1060 patients) ∗∗ [34] If within Green área 5-year recurrence-free survival: 87.4% (1187 patients) [28]∗∗

#### 2.11.2 University of California San Francisco (UCSF)

Other centers around the world started their own HCC - LT protocols. In 2001. Yao et al. assessed 70 patients with cirrhosis and HCC who undervent orthotopic liver transplantation over a 12-year period, at the University of California, that developed their own criteria: a single tumor 6.5 cm or less or 3 or fewer, the largest of which is 4.5 cm or less with a total diameter of 8 cm or less [27] (Table 3). Overall, the UCSF criteria is a modest expansion of the existing Milan Criteria, and significant overlap inevitably exist between the two criteria [33].

#### 2.11.3 Up-to-seven

Mazzafferro et al. revisited the Milan criteria, incorporating 1556 patients to a new study where they developed the ‘‘Up to 7 criteria” this means up to 7 tumors in number the largest of which is less than 7 cm with similar outcomes than the Milan Criteria [34] (Table 3).

#### 2.11.4 Extended Toronto Criteria (ETC)

In response to imaging only criteria, in 2011, Dubay et al. proposed the Toronto Criteria (Table 3), which incorporated 294 patients in a retrospective study, add imaging findings to rule out vascular invasion, using preoperative biopsy pathology, that developed their own criteria: No tumor size or nomber limit, no systemic spread or vascular invvolvement and not poorly differentiated on biopsy (if exceeds Milan) [28].

#### 2.11.5 Metroticket 2.0 (MT2)

In the context of liver transplantation for patients with hepatocellular carcinoma, prediction models are used to ensure that the risk of recurrence after transplantation is acceptably low. The Metroticket 2.0 model has been proposed in 2018 for Mazaferro et al. in a multicenter study with 1018, they performed multivariable competing-risk regression analysis to identify factors associated with HCC-specific death of patients who underwent LT. as an accurate predictor of “tumour-related death” after liver transplantation. They developed a model based on level of AFP, tumour size and tumour number, to determine risk of death from HCC-related factors after liver transplantation [26,29,34] (Table 3).

## 3 Results

Were searched 29 articles (Fig. 2): 2 meta-analysis, 3 randomized clinical trial, 7 review, 17 cohort study: propective (5) and retrospective (12).

Selected studies prior to 2016 were used for historical context. A comprehensive quality assessment of all included studies was performed and synthesis of results was achieved by narrative review.

According to the analysis for Sapisochin et al. [35] report that Milan criteria (MC) has 4-years overall survival of 75%−85% [26,34,[35], [36], [37]], and Alim et al. [32] 5-years overall survival of 70%–75.6% [38], also Toniutto et al. [30] report that disease-free recurrence in 5-years of 83% [26,36], Sapisochin et al. [35] disease-free recurrence in 4 years of 92%; Linkemann [39] disease-free recurrence in 3 years of 74%.

UCSF according for Sapisochin et al. [35] report 73%–87% survival [34,26,[36], [37], [39]], and a disease-free recurrence in 5 years of 80%–90.9% [26,35,39,30], besides Unek et al. [40] report survival of 53.6%.

According to Bento de Souza et al. [41] in a meta-analysis report overall survival MC of 72% and 75%, respectively.

Toronto Criteria according to Toniutto et al. [30] disease-free recurrence in 5 years of 66%–70% [[26], [29], [35], [36], [37]], Linkemann et al. [39] reports 78% disease-free recurrence in 5 years and Falk et al. [31] report 68% [39] of survival and 30% a disease-free recurrence in 5 years, while Ruch et al. [26] reports 66%.

Up-to-7 according to Mazaferro et al. [[26], [29], [35], [36], [37],^42^] report a survival of 65%–71.2%, and Lyu et al. [43] report in an unicentric retrospective study of 96.1% and a disease-free recurrence in 5 years of 64.1%, respectively. The institution of a protocol for preoperative biopsy to exclude patients with poorly differentiated tumors and bridging therapy saw an improvement in survival: from a 5-year survival rate of 61%–79%, and a similar increase in the 5-year disease-free survival: 58%–76% [44].

Lozanovski et al. 32 study that Metroticket 2.0 (MT2) has a survival of 78%–81% [34,29,37], however, disease-free recurrence has not been assessed. MT2 demonstrate that a combination of AFP and tumor burden parameters predicts post-LT outcome far betther than tumor alone [42].

Higher pre-LT alpha-fetoprotein (hazard ratio [HR]1.01; *P* = 0.001), poorly differentiated tumors (HR 13; *P* = 0.039), the presence of microvascular invasion (HR 7.9; *P* = 0.001), higher Total tumour volume (HR 1.03; *P* = 0.001), and faster tumor growth (HR 1.09; *P* = 0.001) were significantly associated with the risk of recurrence [45].

Particularly, the author Constentin et al. [46] reports that EASL and AASLD “remain stagnant because of lack of consensus and limited prospective data. Therefore, we strongly encourage countries moving away from Milan Criteria to carefully collect prospective data on the outcomes of using newer criteria for selecting patients with HCC for LT to overcome current limitations and pave the way to a new international consensus conference dedicated to LT for HCC”.

Patients beyond the Milan criteria with tumor growth 1.61 cm^3^/mo experienced less recurrence (11% vs. 58%; *P* = 0.023) than those beyond the Milan criteria with tumor growth 1.61 cm^3^/mo. Similarly, rate of tumor growth predicted HCC recurrence in those beyond the UCSF criteria [45].

### 3.1 Analysis of distribution of publications and citations

**Fig. 3:**
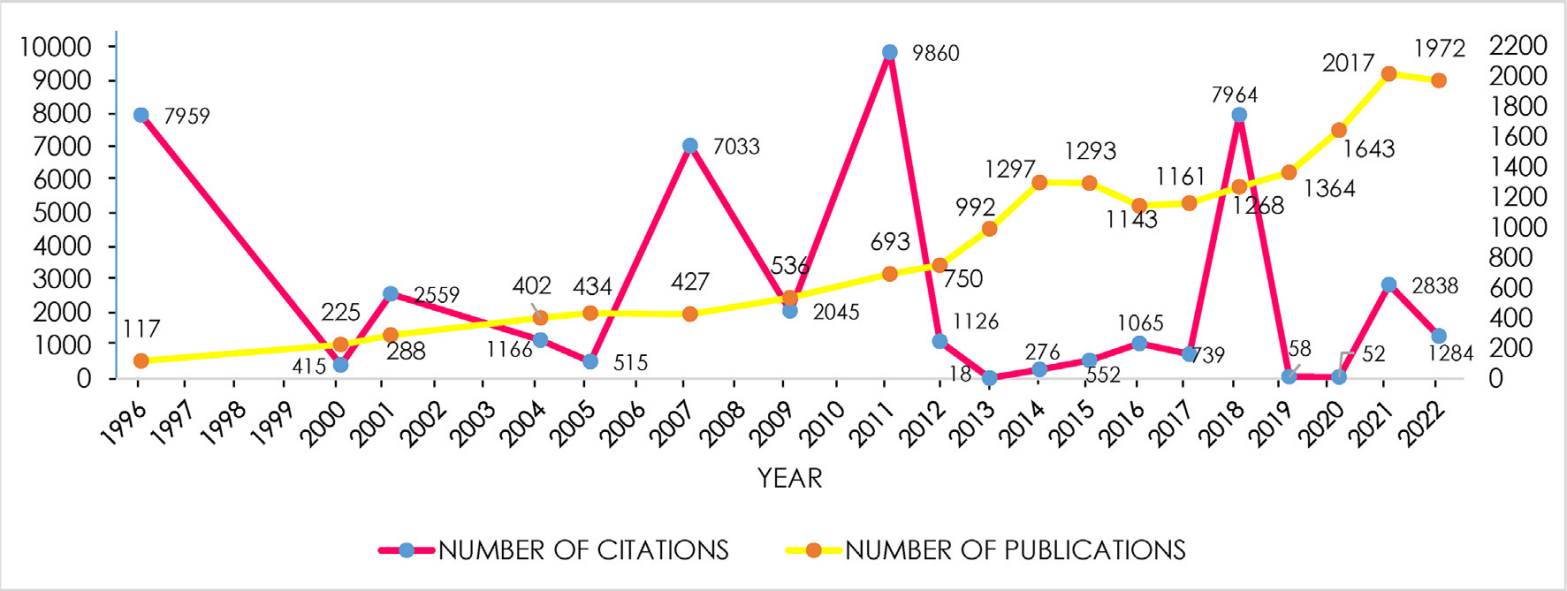
Analysis of distribution of publications and citations.

Based on manual screening with patience, there were 56 publications interrelated to prognostic role of selection criteria for liver transplantation in patients with hepatocellular carcinoma from 1996 to 2022. As illustrated in Fig. 3, the research plotted the number of publications and citations per year, which carried out mathematical function fitting for the curves of annual number of volume and times cited. Specifically, the quantity of articles had increased since 117 in 1996, culminating the peak in 2021 with a total of 2017, with a non-significant decrease during 2022.

### 3.2 Analysis of distribution of countries/regions

The world map indicated scientific output of countries with contribution hepatocellular carcinoma research. As the map of the world shown, among all 17 countries from which publications of prognostic role on prognosis of selection criteria for liver transplantation in patients with hepatocellular carcinoma originated, the Spain, followed by Italy and United States were the leading countries in this field, with articles came out, accounting for 46.4% of the whole. Of note, the most of top productivity countries belong to developed countries. This phenomenon reveals that the number of articles is intimately bound with economic development.

**Fig. 4:**
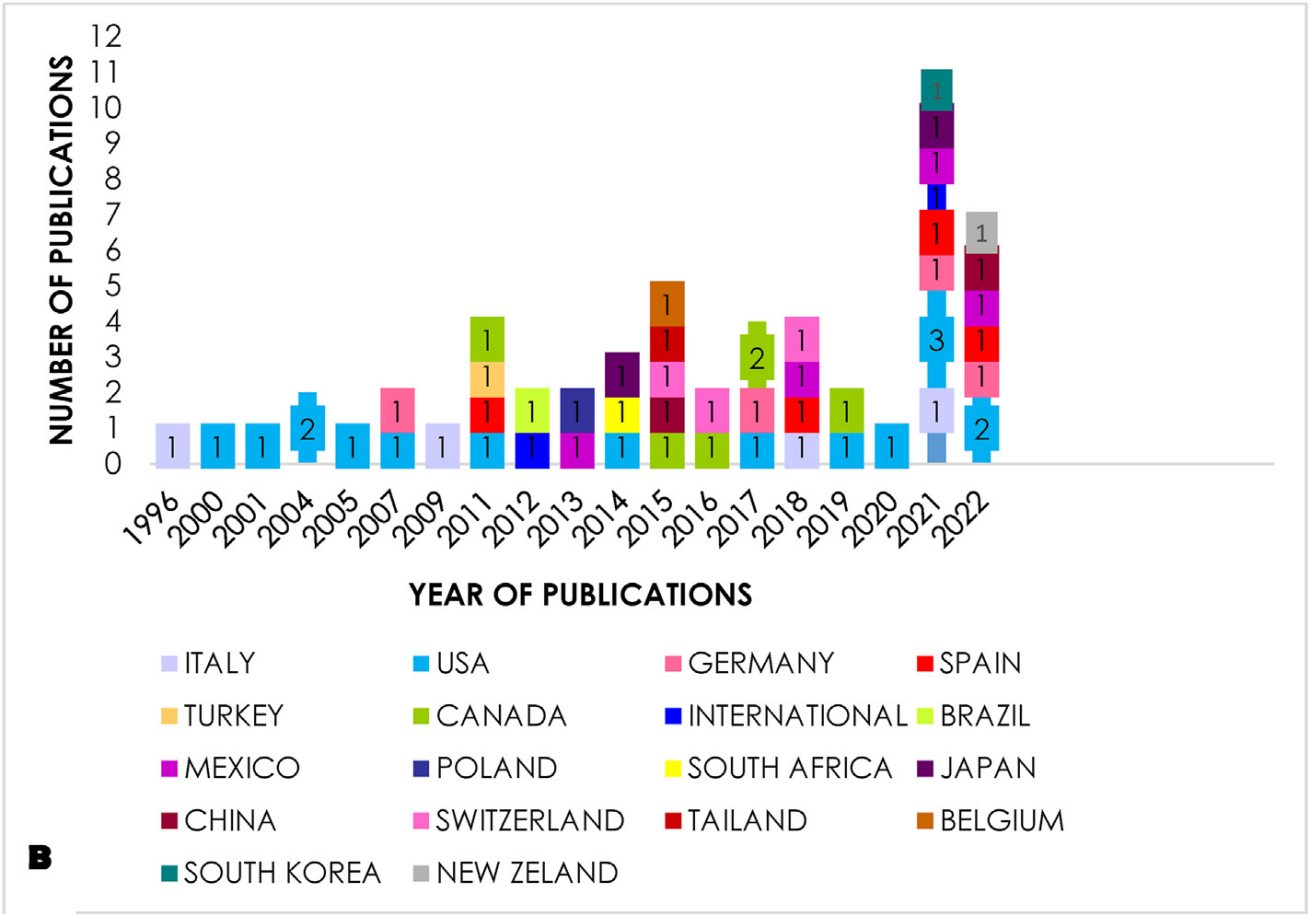
The most of top productivity countries belong to developed countries. This phenomenon reveals that the number of articles is intimately bound with economic development.

A bit farther on are Fig. 4, Table 4, Table 5, we compared the trends in the number of articles between countries by year. It has been noticed that the United States is always the most energetic country in this domain. The continent most interested in this topic is Europe, with a total of 36,780 citations, nearly 6 times more than the American continent. However, we find that occasionally, international studies with a significant impact of 3159 citations in total are conducted.

**Table 4:** Country with total number of citations.

Country	Total number of citations
Canada	1,141
USA	5,802
Mexico	15
Brazil	47
Italy	10,886
Germany	6,456
Spain	14,168
Turkey	46
Poland	13
Switzerland	5,174
Belgium	37
South Africa	132
Thailand	114
China	7
Japan	317
South Korea	7
New Zealand	3
International	3,159

**Table 5:** Continent with total number of citations.

COUNTRY	TOTAL CITATIONS
AMERICA	6611
EUROPE	36,780
AFRICA	132
ASIA	445
OCEANIA	3
INTERNATIONAL	3159

### 3.3 Analysis of distribution of journals

The citing journals related to hepatocellular carcinoma research mainly pertained to surgery, medicine, medical, clinical, molecular, biology and transplantations journals. Therefore, prognostic role of selection criteria for liver transplantation in patients with hepatocellular carcinoma is closely connected with basic and clinical subjects, and the construction of multidisciplinary management needs to be further strengthened in the future.

Table 6 ranks the top 10 most prolific prognostic role of selection criteria for liver transplantation in patients with hepatocellular carcinoma-related journals. The journal with the first four related to prognostic role of selection criteria for liver transplantation in patients with hepatocellular carcinoma was the Lancet gastroenterology and hepatology, followed by New England Journal of Medicine (NEJM), the nature reviews gastroenerology and hepatology and The Lancet Oncology with a total of 27 articles. The two journals, The Lancet gastroenterology and hepatology and NEJM were the highest impact factors and citations, focusing principally on prognostic role of selection criteria for liver transplantation in patients with hepatocellular carcinoma. These outcomes disclose that the majority of articles are published in authoritative of Science Journal Citation Reports (JCR). In terms of JCR 2019 standard, the first 10 journals were principally situated in Q1 partition. These outcomes disclose that the majority of articles are published in authoritative journals, suggesting is attracting the attention of most scientists around the world. Moreover, combined with the country of publications and top productivity journals, it's not too difficult to detect the United Kingdom is in leading position.

**Table 6:** Top 10 most prolific prognostic role of selection criteria for liver transplantation in patients with hepatocellular carcinoma-related journals.

RANK	JOURNAL	IMPACT FACTOR	COUNTRY	CITATION	QUARTILE IN CATEGORY
1	The Lancet gastroenterology and hepatology	168.9	United Kingdom	6145	Q1
2	NEJM	158.5	USA	133956	Q1
3	The nature reviews gastroenerology and hepatology	65.1	United Kingdom	10502	Q1
4	The Lancet Oncology	51.1	United Kingdom	19716	Q1
5	Clinical Gastroenterology and Hepatology (AGA)	33.883	United Kingdom	9256	Q1
6	Journal of hepatology (EASL)	30.083	Netherlands	16924	Q1
7	Ca-A Cancer journal for clinicians	13.4	USA	30318	Q1
8	Molecular cancer (BMC)	11.8	United Kingdom	17050	Q1
9	American journal of transplantation	9.36	United Kingdom	8931	Q1
10	Annals of surgery	9	USA	11322	Q1

## 4 Discussion

Organ allocation for patients in the liver transplant waiting list, assignation is based upon the Model for End stage Liver Disease (MELD) a statistical model based on predicted survival in patients with cirrhosis. MELD criteria do not predict the risk of death among patients with chronic liver disease associated with HCC.

Milan Criteria, based on the evidence of overall survival along with MT2 and disease-free recurrence in 5 years (Table 3), however, they tend of to be very restrictive; a major shortcoming of all currently proposed criteria is that they determine the risk of HCC recurrence after LT based on tumor morphology rather than biology, despite diagnosing HCC within the Milan criteria due to organ shortage, with the time elapsed to find a donor, these criteria are exceeded.

HCC patients meeting the MC have achieved satisfactory 5-year post-transplant survival over the past two decades of LT, which is similar to that observed for benign liver diseases [47].

All assessments of expansion of tumour burden and serum level of AFP show that exceeding Milan criteria is associated an increased prevalence of parameters linked to augmented risk of recurrence [[48], [49], [50]] (microscopic vascular invasion or satellites) with HRs ranging from 3.2 to 4.5 that were even higher than HR of 2.6 for being outside the MC [51] and thus impaired long-term outcome [[48], [49], [50]].

MT2 model had the best outcomes confirming that not only tumor morphology, but also tumor biology should be considered when selecting patients with HCC for transplantation; it has the best 5-year overall survival, howewer, disease-free recurrence has not been assessed.

The UCSF criteria offer the potential benefit of OLT to an additional 5%–20% of patients with HCC who would have otherwise been excluded from OLT under the more restrictive MC [52].

The use of Up-to-seven criteria can be a helpful guidepost for when to consider systemic therapy alone or in addition to TACE [53].

However, liver transplantation is theoretically the best treatment option available today for HCC because it results in the widest possible resection margins for the cancer, removes the remaining liver tissue that is at risk for developing de novo cancer, and restores the hepatic function [54]; even though there are many criteria for liver transplantation, LT still has the best survival than not perfoming LT.

Expanding the indication for living donor LT for HCC will provide a potential curable procedure to patients who otherwise have limited treatment options [55].

Bibliometrics was widley used to process quantitive and visual analysis scientific outputs in biomedical field, which not merely provided the basis for the diagnosis and treatment of diseases, but also forecast the direction of future of development. In this trial, we systematically investigated and summarized the distribution characteristics and research frontiers related to hepatocellular carcinoma from 1996 to 2022 to reveal the current research trend of hepatocellular carcinoma. A growing number of publications have been emerged since the 20th century, especially in the United States and Italy. The collaboration analysis demonstrated that there was limited collaboration between institutions and countries. Moreover the prognosis defined by subtypes and bioinformatics is currently hotspots and highlights. Our study offered an integral overview of hepatocellular carcinoma, particularly for research focus and future directions, which can further accurately guide scholars on liver transplantation and prognostic role of selection criteria for liver transplantation in patients with hepatocellular carcinoma.

Furthermore, as time goes on, the number of publications has grew exponentially, while the number of citations has remained constant, this indicates that research on prognosis of selection criteria for liver transplantation in patients with hepatocellular carcinoma is under the spotlight, as it is the sixth leading cause of death worldwide. In summary, we believe there will be a greater outlook for prognostic of selection criterio for liver transplantation in patients with hepatocellular carcinoma research in the coming years.

Europe is the continent with the highest total number of citations. Spain is the country that contributes the most to scientific communication in this case. In addition, the United States is the country that publishes the most year after year. The diferents journals requires more collaboration between authors, between institutions and countries.

## 5 Conclusion

With the present review, we concluded that the best criteria for HCC are the Milan Criteria, based on the evidence of overall survival along with MT2 and disease-free recurrence in 5 years, however, they tend of to be very restrictive, despite diagnosing HCC within the Milan criteria due to organ shortage, with the time elapsed to find a donor, these criteria are exceeded.

MT2 model had the best outcomes confirming that not only tumour morphology, but also tumour biology should be considered when selecting patients with HCC for transplantation; it has the best 5-year overall survival, howewer, disease-free recurrence has not been assessed.

This study has analyzed systematically and summarized the distribution characteristics and research frontiers by bibliometrics concerning prognostic role of selection criteria for liver transplantation in patients with hepatocellular carcinoma the collaborations are sufficient to reach a consensus that the Milan criteria are the best criteria.

## Funding

The author declares no funding has been received for this paper.

## Author contributions

- Conceived and designed the analysis: Pamela Scarlett Espinoza Loyola, Omar Vásquez Gómez, Isidoro Aczel Sánchez-Cedillo and Víctor Jose Visag Castillo.

- Wrote the paper: Pamela Scarlett Espinoza Loyola.

- Contributed data or analysis tools: Diana Laura Muratalla Bautista, Carla Escorza-Molina and Luis Jorge Fernández López.

- Collected the data: Karen Adela Hernández Bautista, Elizabeth Gil White, José Antonio González Moreno, Víctor Manuel Páez Zayas and Paul Santiago Mogrovejo Vázquez.

- Performed the analysis: Daniel Angel Torres del Real, Pamela Scarlett Espinoza Loyola, Omar Vásquez Gómez, Isidoro Aczel Sánchez-Cedillo and Víctor Jose Visag Castillo.

- Created the images and visual resources: Fernando Mondragón Rodríguez.

## Acknowledgments

Words cannot express my gratitude to Victor Visag Castillo and Omar Vasquez Gomez for their invaluable patience and feedback. I would like to extend my sincere thanks to Aczel Sanchez Cedillo for encourage scientific communication.

## Declaration of competing interest

The author declares there are no conflicts of interest.

## Data available statement

Data supporting this study are included within the article and/or supporting materials.

## Ethics statement

### Protection of human and animal subjects

The authors declare that no experiments were performed on humans or animal for this study.

### Confidentiality of data

The authors declare that they have followed the protocols of their work center on the publication of patient data.

### Right to privacy and informed consent

The authors declare that no patient data appear in this article.

## Informed consent

We do not require an informed consent.
